# P-1927. Testing for Coccidioidomycosis in Hospitalized Patients with Pneumonia within the Coccidioides-endemic Area

**DOI:** 10.1093/ofid/ofaf695.2096

**Published:** 2026-01-11

**Authors:** Janis E Blair

**Affiliations:** Mayo Clinic Hospital, Phoenix, Arizona

## Abstract

**Background:**

Within the *Coccidioides*-endemic area, coccidioidomycosis (cocci) has been identified as the cause of pneumonia in 15-29% of patients with community-acquired pneumonia (CAP). Although efforts to improve testing CAP patients for cocci are underway in the outpatient settings in Arizona, we do not know whether testing is common among patients admitted to the hospital with pneumonia. We undertook the current study to assess the frequency of testing for cocci among hospitalized patients with pneumonia in our institution, and to understand whether positive serologic testing led to a reduction in antibiotic use.

**Methods:**

We commissioned the creation of an electronic tool by our Information Technology colleagues to create a report to identify inpatients of Mayo Clinic Hospital with a pneumonia diagnosis (J18.0 – J18.9) and information regarding testing for *Coccidioides* by any serology (enzyme immunoassay, immunodiffusion or complement fixation) and patient receipt of antibiotics or antifungal treatment (name, inclusive dates).

**Results:**

Between January 1, 2024 and December 31, 2024, 1162 patients were admitted to the Hospital Internal Medicine service at Mayo Clinic Hospital, Phoenix AZ with a pneumonia diagnosis. Among these, 771 (66%) were tested serologically for coccidioidomycosis, and 87/771 (11%) were found to be positive. By calendar month, the percent of patients tested ranged 59 – 73%, and percent test positivity ranged 3% (March) - 19% (December). The mean time from admission to test order was 0.5 days (range 0 – 6 days) and mean time from admission to positive result was 3.0 days (range 0.4 – 12.0 days). However, among seropositive patients, the duration of antibiotics was 4.2 days, not different when compared with patients who were either seronegative or not tested, (4.1 days, p=NS). 173 (14.9%) received antifungal treatment during hospitalization, among whom 55 (31.2%) had positive coccidioidal serology and 118 (68.2%) did not.

**Conclusion:**

Coccidioidomycosis is an important cause of pneumonia within the inpatient setting. However, one third of patients were not tested, representing an opportunity to understand and overcome barriers to testing. Positive tests were not associated with a reduction of antibiotic use.

**Disclosures:**

All Authors: No reported disclosures

